# 

**Published:** 1991-12

**Authors:** 


### WEST OF ENGLAND MEDICAL JOURNAL

**DECEMBER 1991** **Volume 106(iv)**

Copyright © West of England Medical Journal Ltd 1991.

**CONTENTS**

IFC **Index for 1991.**
90 **Editorial**
91 **Who wrote the Hippocratic Oath.** Ian Bailey
92 **The Unwrapping of the Bristol Mummy.** J. Sluglett
94 **The Care of the Newborn in Antiquity.** Beryl Corner
95 **Epilepsy, Brain and Mind.** Jonathan Bird
96 **Captain Thomas Dover.** John Crossley
98 **Childbirth, Lessons from the past.** Peter Dunn
99 **The Leonardo Anatomical Drawings.** Michael Wilson
101 **Caleb Hillier Parry.** Brian Jones
103 **The American Prisoners in Dartmoor 1813-1815.** Peter Trafford
105 **Pathos and Pathology in the life of Lord Byron.** Roger Celestin
106 **Serendipity and Logic in Medical Research.** Brandon Lush
107 **Paul Ehrlich and Almroth Wright.** William Gillespie
108 **Archibald Menzies, Surgeon Botanist.** John Naish
110 **History of the Medical Services in Weston-super-Mare.** Huw Roberts
111 **Lunacy Bristol Fashion.** Donal Early
112 **The Burdens and Bristol.** Jose Jancar
115 **From our Correspondents.** Notes from Cornwall, How Basic can you get? New Senior Lecturer in Transplantation at Southmead
116 **Letter to the Editor.** **The Cornwall Helicopter**
117 **Obituary.** Herbert Kitchener Bourns
118 **Book Reviews.** Clinical Oncology, 80 Years in Bath, The return of blood to the Heart
119 **South West Orthopaedic Club.**

